# Mechanisms by Which Exercise Delays Brain Aging Through Regulation of the Mitochondrial Quality Control System

**DOI:** 10.3390/biology15110854

**Published:** 2026-05-29

**Authors:** Xinyi Zhu, Lei Shi, Yahong Dong, Yingjie Sun, Qiguan Jin

**Affiliations:** College of Physical Education, Yangzhou University, Yangzhou 225000, China

**Keywords:** brain aging, exercise intervention, mitochondrial dysfunction, mitochondrial quality control, mitochondrial autophagy, neuromuscular axis

## Abstract

Brain aging is a complex biological process characterised by progressive neuronal and synaptic decline, in which disruption of mitochondrial quality control plays a central role. This system encompasses multiple synergistic components, including mitochondrial biogenesis, dynamic equilibrium, autophagic clearance, and energy metabolism. Aging induces dysfunction across these processes, precipitating mitochondrial fragmentation, functional decline, and energy crises, ultimately driving cognitive deterioration. Exercise is a promising non-pharmacological intervention for preserving brain health during aging, and its benefits may be mediated, at least in part, through modulation of mitochondrial quality control. Specifically, exercise has been shown to activate key signaling pathways such as AMPK/SIRT1/PGC-1α, thereby promoting mitochondrial biogenesis and metabolic adaptation. It may also regulate mitochondrial dynamics and mitophagy via pathways including cAMP/PKA/Drp1 and AMPK/mTOR. In addition, emerging evidence indicates that exercise may influence brain mitochondrial function through activity-dependent regulation of mitochondrial gene expression and systemic signaling factors. Furthermore, this review discusses potential differences between exercise modalities and highlights future directions for personalised intervention strategies, providing a theoretical basis for the application of exercise in delaying brain aging and preventing neurodegenerative diseases.

## Simple Summary

Brain aging is a gradual process that leads to a decline in memory, learning ability, and overall cognitive function. A key factor underlying this process is the dysfunction of mitochondria, the structures responsible for producing energy inside brain cells. When mitochondrial function deteriorates, brain cells face energy shortages and increased damage, leading to faster neuronal loss and cognitive impairment, which accelerates neuronal degeneration and cognitive decline. Growing evidence shows that physical exercise is a powerful non-drug strategy to protect the aging brain. Regular exercise can improve mitochondrial health, enhance energy production, and support the brain’s ability to adapt to stress and aging-related changes. This review summarises current research showing how exercise helps maintain mitochondrial function, supports brain plasticity, and slows age-related cognitive decline. It also discusses how different types of exercise may have distinct benefits and highlights the potential of personalised exercise strategies for brain health. These findings provide a scientific basis for using exercise as an effective approach to promote healthy brain aging and to support the prevention and treatment of neurodegenerative diseases.

## 1. Introduction

The global population is aging at an unprecedented rate, with those aged 65 and over projected to exceed 1.5 billion by 2050 [1]. Age-related cognitive decline has emerged as a significant public health challenge [2]. Core hallmarks of brain aging include volume reduction in key regions such as the hippocampus, diminished synaptic plasticity, weakened neurogenesis, and “inflammaging” characterised by chronic low-grade inflammation. Collectively, these alterations lead to progressive decline in learning, memory, and executive functions [3].

Mitochondria, serving as the central hub for neuronal energy metabolism and signal integration, are essential for maintaining neuronal survival, synaptic transmission, and neuroplasticity [4,5]. Recent research indicates that brain aging is closely associated with progressive disruption of the mitochondrial quality control system rather than a single molecular event [4,5,6]. This system includes mitochondrial biogenesis, mitochondrial dynamics (fusion and fission), mitophagy, and energy metabolism. During aging, impairments in these interconnected processes promote the accumulation of dysfunctional mitochondria, excessive reactive oxygen species (ROS) production, disrupted calcium homeostasis, and reduced ATP generation, thereby contributing to neuronal dysfunction and neurodegeneration [4,5,6].

Despite ongoing advances in drug development targeting individual pathways, their efficacy often remains limited. In contrast, regular exercise training is widely recognized as an effective non-pharmacological strategy for improving cognitive function and brain structure in aging individuals [7,8]. Its benefits may extend beyond those of single-target drugs, as it may modulate multiple signaling pathways to coordinate responses to multidimensional aging characteristics [8]. Existing evidence suggests that exercise’s promotion of brain health may be rooted in multi-targeted, systemic remodelling of the mitochondrial quality control system [9,10]. However, how exercise coordinately regulates the different components of this network, their upstream signaling hubs, and emerging systemic mediators remains incompletely understood. This review adopts an integrated perspective centred on mitochondrial quality control to examine how exercise may delay brain aging and to outline directions for mechanism-based, personalised interventions.

## 2. Materials and Methods

This article is a narrative review. Literature searches were conducted in PubMed, Web of Science, and Scopus databases for studies published in English up to January 2026. Keywords included combinations of “brain aging”, “mitochondrial dysfunction”, “mitochondrial quality control”, “exercise”, “mitophagy”, “mitochondrial dynamics”, “mitochondrial biogenesis”, and “cognitive decline”.

Original research articles, clinical studies, and relevant review articles related to brain aging, mitochondrial function, and exercise interventions were considered. Priority was given to recent studies and representative mechanistic investigations in both animal models and human studies. This review was conducted as a narrative review rather than a systematic review; therefore, a formal systematic review protocol was not applied; instead, the selected literature was synthesized to provide an updated overview of the mechanisms linking exercise, mitochondrial quality control, and brain aging.

As this study is a narrative review based exclusively on published literature, no experimental chemicals, reagents, devices, or commercial biological materials were used. References were managed using EndNote 21 (Clarivate Analytics, Philadelphia, PA, USA).

## 3. Characteristics of Brain Aging

### 3.1. Macro-Structural Features

The most prominent manifestation of brain aging at the macro-structural level is the progressive reduction in brain tissue volume and weight. Research indicates that between the ages of 20 and 60, brain weight decreases by approximately 0.1% annually on average. After the age of 70, the rate of atrophy may accelerate, with an average annual weight reduction of 2 to 5% [11]. Cognitive decline accompanies atrophy across the entire brain or in specific regions, manifesting in reduced processing speed, working memory, reasoning, and executive function [12]. Crucially, this atrophy is not uniformly distributed but follows specific patterns. Through deep learning analysis of nearly 50,000 brain scans, researchers have identified five distinct patterns of brain atrophy. These patterns correlate with specific aging trajectories and disease risks. Different combinations of atrophy patterns can predict the risk of diseases such as Alzheimer’s and Parkinson’s, as well as the transition from normal cognition to mild cognitive impairment [13]. Among these, the frontal and temporal lobes (particularly the hippocampus) exhibit the most pronounced atrophy and are closely associated with higher cognitive functions such as memory and executive function [14,15].

### 3.2. Microscopic Characteristics

At the microscopic level, aging triggers a series of complex cellular and molecular events.

#### 3.2.1. Synaptic and Neural Circuit Remodelling

Synapses, as key structures for neural information transmission, undergo age-related alterations that directly impact brain function. Brain aging manifests as reduced synaptic density and diminished dendritic spines, impairing synaptic plasticity. Neurogenesis in the subventricular zone and hippocampal dentate gyrus significantly declines, representing primary drivers of age-related cognitive decline [16,17]. Studies in the hypothalamus reveal a substantial decline in synaptic density of approximately 28% from youth to old age. Earlier alterations emerge in middle age, characterised by enhanced interneuronal connections within inhibitory (GABAergic) neural circuits and prolonged activity zones in excitatory synapses. This may disrupt the excitatory–inhibitory balance within neural networks, compromising the precision of information processing [18].

#### 3.2.2. Cellular Senescence and Energy Crisis

Aging neurons and astrocytes frequently exhibit hallmarks of cellular senescence: increased volume, flattened morphology, heightened lysosomal activity characterised by elevated senescence-associated β-galactosidase (SA-β-gal) activity, and presentation of a senescence-associated secretory phenotype (SASP) [19,20], accompanied by increased expression of cell cycle arrest proteins (such as p16, p21), oxidative stress, and accumulated DNA damage [21]. Of particular significance is the decline in neuronal energy supply systems. A groundbreaking 2024 study in *Science* revealed that the coupling mechanism driving neuronal activity-mediated mitochondrial DNA transcription (E-TC_mito_) weakens with age, resulting in insufficient local energy generation near synapses. These findings suggest that activity-dependent mitochondrial transcription may contribute to cognitive resilience during aging, although extrapolation to humans remains premature [22]. However, brain aging exhibits cell-type and brain-region specificity, and the precise molecular mechanisms underlying this phenomenon require further investigation [20].

#### 3.2.3. Protein Homeostasis Dysregulation and Inflammation

Brain aging is accompanied by extensive alterations in protein expression. A recent plasma proteomics study identified 13 proteins associated with brain biological age and reported non-linear trajectories with inflection points around 57, 70, and 78 years, suggesting stage-specific features of brain aging. The peak around age 70 shows the strongest association with brain structural atrophy and dementia risk, while the peak around age 78 correlates with the activation of inflammatory pathways such as JAK-STAT, highlighting the central role of neuroinflammation in late-stage aging [23]. In parallel, the establishment of an “inflammaging” microenvironment—partly driven by SASP—promotes sustained release of pro-inflammatory mediators, exacerbating neuronal dysfunction [24]. Supporting this, immune-related genes (particularly complement system genes) have been identified as robust biomarkers of brain aging and show inverse correlations with synaptic function-related genes [25,26]. This suggests that age-related immune activation may contribute to synaptic decline.

### 3.3. Changes in Neural Functional Networks

At the systemic level, neural network activity in the aging brain exhibits characteristic alterations. A magnetoencephalography (MEG) study spanning ages 18 to 88 revealed that aging alters the microstate patterns of spontaneous neural oscillations. This manifests as shorter durations but increased frequency of alpha rhythms (8–13 Hz) associated with sensory processing microstates, potentially reflecting diminished sensory information processing efficiency and compensatory attempts. Alterations in theta and beta rhythms within specific microstates may correlate with age-related declines in motor function [27].

In summary, brain aging constitutes a non-linear, phased dynamic process exhibiting distinct characteristics across different life stages. The following section discusses how dysregulation of the mitochondrial quality control system underlies these age-related changes.

## 4. Brain Aging and Dysregulation of the Mitochondrial Quality Control System

**Figure 1:**
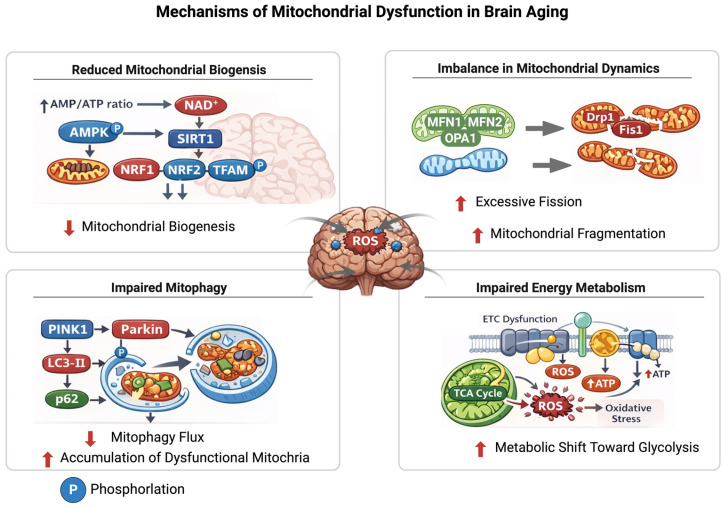
Schematic illustration of mitochondrial quality control disruption during brain aging. The mitochondrial quality control system includes mitochondrial biogenesis, mitochondrial dynamics (fusion and fission), mitophagy, and energy metabolism. Aging impairs mitochondrial biogenesis, promotes excessive mitochondrial fission and fragmentation, reduces mitophagy efficiency, and disrupts cellular energy metabolism. These alterations lead to the accumulation of dysfunctional mitochondria, increased reactive oxygen species (ROS) production, and reduced ATP generation, ultimately contributing to neuronal dysfunction, synaptic impairment, and cognitive decline. Red upward arrows indicate increased activity or expression, whereas red downward arrows indicate decreased activity or expression. Gray directional arrows indicate signaling flow, mechanistic progression, or causal interactions among pathways. Blue circles labeled “P” denote phosphorylation. Created with BioRender.com.

This research indicates that with advancing age, gene expression in the human brain predominantly shifts towards downregulation [28]. Genes downregulated in the brain are associated with mitochondrial function, synaptic plasticity, inhibitory interneurons, and the ubiquitin–proteasome system (UPS). Conversely, genes related to stress responses, immune/inflammatory responses, metal ion homeostasis, myelin-associated functions, and glial cells tend to be upregulated [28]. The mitochondrial quality control system is central to maintaining neuronal energy and health. The mitochondrial dysfunction observed with aging is not an isolated event but rather the consequence of the collapse of its intrinsic quality control network (Figure 1), forming the cornerstone of brain aging.

### 4.1. Brain Aging and Mitochondrial Biogenesis

Mitochondrial biogenesis is the process of generating new mitochondria, primarily regulated by the peroxisome proliferator-activated receptor gamma coactivator-1α (PGC-1α) and its upstream modulators AMP-activated protein kinase (AMPK)/sirtuin 1, alongside downstream effectors nuclear respiratory factor 1/2 (NRF1/2), the estrogen-related receptor alpha (ERRα), and mitochondrial transcription factor A (TFAM) [29,30]. Under physiological conditions, AMPK and SIRT1 coordinately activate PGC-1α, which then promotes the transcription of mitochondrial genes through NRF1/2, ERRα, and TFAM, thereby supporting mitochondrial biogenesis. In the aging brain, impaired AMPK responsiveness, reduced sirtuin activity, weakened TFAM–mtDNA binding, and cumulative mtDNA damage collectively compromise mitochondrial biogenesis, thereby contributing to neuronal bioenergetic failure [31,32,33,34]. Although TFAM expression may increase in aged cerebral cortex, its binding to mitochondrial DNA (mtDNA) is markedly reduced, suggesting that compensatory upregulation is insufficient to preserve effective mitochondrial biogenesis [35]. In parallel, age-related accumulation of mtDNA mutations and deletions further compromises mtDNA replication and oxidative phosphorylation capacity [6,36,37]. Together, impairment of the AMPK/SIRT1/PGC-1α signaling axis, reduced TFAM–mtDNA interaction, and progressive mtDNA damage constitute a major molecular basis for defective mitochondrial biogenesis in the aging brain, thereby contributing to neuronal bioenergetic failure and functional decline [38]. In addition, the age-associated reduction in NRF2 may further aggravate mitochondrial vulnerability, highlighting NRF2-related pathways as potential targets for delaying brain aging [39]. In addition to these canonical pathways, recent evidence suggests that serum response factor (SRF) may modulate age-related mitochondrial dysfunction, while earlier studies have shown that the SRF–cofilin–actin signaling axis regulates mitochondrial dynamics [40,41]. However, its role in brain aging, particularly in the context of exercise, remains to be determined.

### 4.2. Brain Aging and Mitochondrial Dynamics

Mitochondrial dynamics constitute a vital process for maintaining mitochondrial morphological and functional homeostasis, encompassing two opposing yet coordinated mechanisms: fusion and fission. Fusion is primarily mediated by mitofusin 1/2 (Mfn1/2) and optic atrophy protein 1 (OPA1), whereas fission depends on dynamin-related protein 1 (Drp1) and its outer mitochondrial membrane adaptors, including mitochondrial fission factor (Mff), mitochondrial fission protein 1 (Fis1), and MiD49/51 [42,43]. Areas of mitochondrial kinetic disorder are prevalent in the aging brain, characterised by reduced expression of fusion proteins such as Mfn2 and OPA1, alongside significantly elevated levels of the fission protein Drp1 (particularly its activated form p-Drp1 S616) and Fis1. This leads to excessive mitochondrial fission and fragmentation [44,45,46]. Concurrently, PUM2, which regulates Drp1 mitochondrial translocation, is upregulated during aging. This further amplifies fission abnormalities by inhibiting Mff protein synthesis [46]. This fragmented mitochondrial network exhibits impaired function, reduced membrane potential (ΔΨm), and suppressed respiratory chain activity. Consequently, synaptic energy supply is compromised, accelerating neuronal aging and cognitive decline [47,48,49,50]. Corresponding to enhanced fragmentation, aging brains exhibit markedly reduced expression of fusion proteins Mfn1, Mfn2, and OPA1. This diminishes mitochondrial network integration capacity, elevates mitochondrial and cytoplasmic ROS levels [48,51,52,53,54], and consequently impairs the maintenance and differentiation of neural stem cells (Sox2+) and newborn neurons (DCX+). This leads to impaired hippocampal neurogenesis and reduced synaptic density [54]. Consequently, the disruption of mitochondrial dynamic equilibrium represents one of the key molecular underpinnings of mitochondrial dysfunction.

### 4.3. Mitophagy and Brain Aging

Mitophagy constitutes a specialised form of autophagy responsible for selectively eliminating aged or damaged mitochondria, serving as a crucial quality control mechanism for removing impaired mitochondria. This process involves the encapsulation of damaged mitochondria within autophagosomes to form double-membrane vesicles, which subsequently fuse with lysosomes for degradation, thereby maintaining cellular energy and metabolic homeostasis [55]. This process is commonly assessed using markers such as LC3, BNIP3L/NIX, Parkin, p62, and Beclin-1 [56,57]. In the aging brain, mitophagy flux is broadly impaired. Compared with young animals, aged brains show reduced levels of Beclin-1 and LC3-II, abnormal accumulation of p62, and attenuated mitophagic activity in regions such as the hippocampal dentate gyrus [58,59,60]. Although the PINK1/Parkin pathway may be up-regulated in the aging brain, its function is severely impaired, leading to the ineffective clearance and progressive accumulation of damaged mitochondria [61,62]. Mitophagy failure may permit the accumulation of damaged mitochondria and mtDNA-driven inflammatory signaling, including cGAS–STING activation, thereby aggravating neuroinflammation [63,64]. It also compromises blood–brain barrier (BBB) integrity and diminishes cerebral vascular health [65]. Thus, the decline in mitochondrial autophagy levels within the brain during aging represents a key mechanism underpinning mitochondrial dysfunction and neuronal functional decline.

### 4.4. Brain Aging and Abnormal Energy Metabolism

Because neurons have exceptionally high energy demands, mitochondrial energy metabolism is indispensable for maintaining membrane excitability, neurotransmitter turnover, and synaptic plasticity [66]. However, this intricate energy production system undergoes systemic decline with advancing age (Table 1), with its dysfunction recognised as a key cellular biological basis driving brain aging and cognitive decline [67]. One major manifestation is the impairment of tricarboxylic acid (TCA) cycle function. The activity or expression of several rate-limiting enzymes, including α-ketoglutarate dehydrogenase, citrate synthase, and malate dehydrogenase, is reduced in the aging brain, thereby limiting the generation of reducing equivalents required for oxidative phosphorylation [68,69]. At the same time, dysfunction of the electron transport chain, reduced mitochondrial membrane potential, and increased electron leakage collectively diminish ATP production while promoting excessive reactive oxygen species generation [66,67]. This creates a vicious cycle of oxidative damage and energetic insufficiency [70,71]. As oxidative phosphorylation becomes less efficient, aging neurons increasingly rely on glycolysis to meet basic energetic demands, reflecting a loss of metabolic flexibility [67,68]. Synapses are particularly vulnerable to this shift because of their high and spatially restricted energy requirements. Local ATP deficiency at axon terminals can impair neurotransmitter release, postsynaptic responsiveness, and synaptic plasticity, ultimately contributing to deficits in learning and memory [69]. Recent evidence further suggests that the coupling between neuronal activity and mitochondrial gene transcription becomes attenuated with age. In young brains, neuronal activity can rapidly stimulate mitochondrial transcriptional responses to match local energy demands, whereas this adaptive mechanism is weakened in aging [22]. This age-related disruption of activity-dependent mitochondrial regulation may represent an additional mechanism linking mitochondrial dysfunction to cognitive decline.

Thus, the energy crisis in brain aging constitutes a multi-layered, systemic failure originating from the decline in core enzyme function within the TCA cycle and oxidative phosphorylation. This leads to insufficient ATP synthesis, excessive ROS production, and ultimately triggers synaptic energy starvation alongside the failure of the “on-demand energy supply” mechanism. Reversing this process requires not only supportive strategies such as supplementing metabolic substrates and employing antioxidants but also focusing on reactivating and enhancing mitochondrial function and adaptability through physical activity (such as exercise) and cognitive engagement. This approach rebuilds a stable and efficient energy supply system for the aging brain.

## 5. Exercise as a Multifunctional Regulator of the Mitochondrial Quality Control System

**Figure 2:**
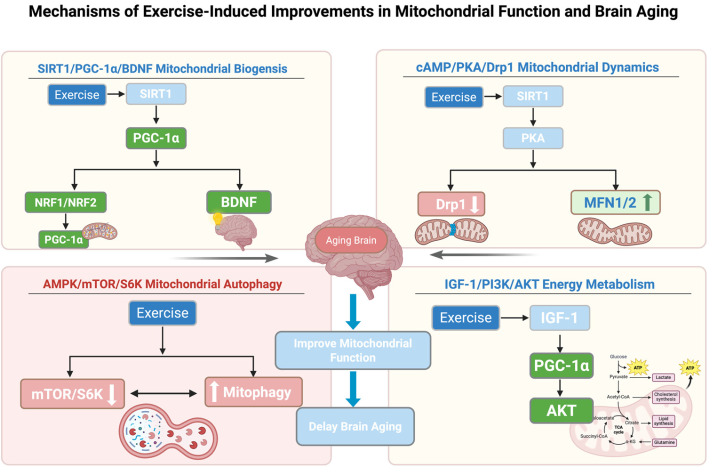
Schematic overview of the mechanisms by which exercise remodels the mitochondrial quality control system in the aging brain. Exercise activates mitochondrial biogenesis through the SIRT1/PGC-1α/BDNF signaling axis, restores mitochondrial dynamics by regulating fusion–fission balance (e.g., Drp1, Mfn1/2, OPA1), enhances mitophagy via AMPK/mTOR-dependent signaling, and improves mitochondrial energy metabolism through the IGF-1/PI3K/AKT axis. These coordinated adaptations improve mitochondrial function, maintain neuronal homeostasis, and ultimately contribute to delayed brain aging and improved cognitive resilience. Created with BioRender.com.

Exercise may improve several aspects of mitochondrial quality control in the aging brain and thereby contribute to neuroprotection (Figure 2).

### 5.1. Exercise Activates Mitochondrial Biogenesis and Enhances Metabolic Resilience

Exercise serves as a potent stimulus for activating mitochondrial biogenesis in the brain (Table 2). Animal studies indicate that prolonged exercise significantly upregulates the expression of SIRT1, PGC-1α, NRF1/2, and TFAM in brain regions such as the hippocampus [79,80,81]. Activation of PGC-1α may further promote the synthesis of brain-derived neurotrophic factor (BDNF), which plays a pivotal role in neural plasticity and neuronal survival [82,83,84]. Through the core signaling axis of AMPK/SIRT1/PGC-1α/BDNF, experimental studies suggest that exercise enhances mitochondrial biogenesis, replenishing the aging brain with functionally competent mitochondria and elevating energy metabolism potential [31,85].

Among diverse strategies countering cerebral aging, exercise training demonstrates irreplaceable advantages, with one core mechanism being its capacity to efficiently activate mitochondrial biogenesis within the brain. For neurons with exceptionally high energy demands, maintaining the vigour of this process underpins their synaptic plasticity, signal transduction, and long-term survival. The age-related decline in mitochondrial biogenesis constitutes a pivotal factor in causing neuronal energy insufficiency and diminished metabolic plasticity (i.e., the cell’s capacity to adapt to fluctuations in energy requirements) [31]. Extensive research indicates that regular exercise represents one of the most effective non-pharmacological interventions for reversing this age-related decline, primarily through the integrated signaling axis of energy sensing–transcriptional regulation–neurotrophic support.

Exercise-induced alterations in cellular energy status (such as elevated AMP/ATP ratios) primarily activate the energy sensor AMP-activated protein kinase (AMPK). Activated AMPK directly phosphorylates or enhances NAD^+^ levels to activate the deacetylase SIRT1, thereby jointly regulating the master regulatory factor peroxisome proliferator-activated receptor gamma coactivator-1α (PGC-1α) [31]. The deacetylation and activation of PGC-1α are crucial steps for its functional engagement. It translocates to the nucleus, where it cooperates with transcription factors such as nuclear respiratory factor 1/2 (NRF1/2), ultimately upregulating the expression of mitochondrial transcription factor A (TFAM). TFAM enters mitochondria, where it governs mtDNA replication, maintenance, and transcription, thereby driving the synthesis of new mitochondria [29,30]. Crucially, exercise-induced PGC-1α activation also directly promotes brain-derived neurotrophic factor (BDNF) synthesis [83]. BDNF serves not only as a key mediator of synaptic plasticity and neuronal survival, but its signaling feedback further supports mitochondrial biogenesis and function, forming a positive feedback loop [82]. Thus, through the AMPK/SIRT1/PGC-1α/BDNF axis, exercise achieves comprehensive remodelling spanning energy sensing, gene expression, functional structures (new mitochondria), and supportive environments (neurotrophic factors). This provides the aging brain with a robust mitochondrial reserve, excelling in both quality and quantity, fundamentally enhancing its metabolic resilience and stress resistance.

In summary, exercise powerfully drives mitochondrial biogenesis in the aging brain by activating the highly integrated AMPK/SIRT1/PGC-1α/BDNF signaling network. This process not only directly increases the number of functionally competent mitochondria, compensating for age-related depletion, but more importantly optimises the neuronal survival and plasticity microenvironment by elevating BDNF levels. Ultimately, exercise intervention endows the brain with enhanced metabolic resilience—the capacity to mobilise and utilise energy more efficiently when confronted with increased energy demands or metabolic stress. This establishes a robust cellular energetics foundation for delaying cognitive decline and sustaining brain health.

### 5.2. Exercise Restores Mitochondrial Dynamics and Mitophagic Flux

In the aging brain, both core pillars of the mitochondrial quality control system—mitochondrial dynamics (the balance between fusion and fission) and mitochondrial autophagy (selective clearance of damaged mitochondria)—exhibit significant dysfunction. Dysregulated dynamics lead to mitochondrial network fragmentation and functional loss, while impaired autophagic flux causes accumulation of dysfunctional mitochondria. Together, these exacerbate oxidative stress and energy crises, driving neuronal degeneration [6,44,55]. Experimental studies suggest that exercise intervention may partially reverse these alterations (Table 3). As a potent physiological stimulus, it synergistically reconfigures the dynamic equilibrium and renewal capacity of the mitochondrial network by activating multiple interconnected signaling pathways, thereby preserving neuronal metabolic health and cognitive function [10].

Mitochondrial morphology and function are highly dependent on the dynamic equilibrium between fusion and fission. Fusion (mediated by Mfn1/2 and OPA1) facilitates exchange of contents, compensates for defects, and maintains network integrity and membrane potential; fission (mediated by Drp1 and its receptors) promotes the isolation, distribution, and subsequent clearance of damaged segments [42,43]. Aging brains commonly exhibit excessive fission (increased Drp1 activity) coupled with insufficient fusion (reduced expression of Mfn1/2, etc.), leading to the accumulation of numerous small, dysfunctional mitochondrial fragments [45,46].

Exercise represents a key non-pharmacological means of restoring this equilibrium. One core mechanism involves activating the cyclic adenosine monophosphate (cAMP)/protein kinase A (PKA) signaling pathway. Exercise may restrain excessive mitochondrial fission partly through cAMP/PKA-dependent modulation of Drp1 phosphorylation, although direct evidence in the aging brain remains limited [86]. This phosphorylation inhibits Drp1 recruitment to mitochondria and its GTPase activity, effectively suppressing excessive mitochondrial fission [87,88]. Concurrently, exercise also upregulates the expression of mitochondrial fusion proteins, such as mitochondrial fusion protein 2 (Mfn2) [89]. This dual regulation—inhibiting fission while promoting fusion—collectively drives the transformation of mitochondria in aging neurons from a fragmented state towards a healthier, interconnected network morphology. This facilitates the stabilisation of mitochondrial membrane potential, optimises oxidative phosphorylation efficiency, and provides a sustained, stable energy supply to synapses [50].

Mitochondrial autophagy represents the terminal quality control step for eliminating dysfunctional mitochondria arising from kinetic imbalances or accumulated damage. The age-associated decline in mitochondrial autophagy correlates with defects across multiple stages of the process, including initiation (e.g., reduced expression of Beclin-1 and LC3-II) and execution (e.g., impaired PINK1/Parkin pathway function and p62 accumulation) [59,61]. Exercise may enhance this mitochondrial “scavenger” function.

The energy expenditure effect of exercise activates the cellular energy sensor AMPK. Activated AMPK initiates autophagy through a dual mechanism: firstly, it directly phosphorylates and activates key components within the autophagy initiation complex; secondly, and more critically, it inhibits the activity of the mammalian target of rapamycin (mTOR) [90]. mTOR serves as the primary regulator of cellular growth and anabolism, whilst also acting as a potent inhibitor of autophagy. Exercise, via AMPK-mediated mTOR inhibition, releases this braking effect on autophagy [91]. This process is particularly evident in the suppression of activity in mTOR’s downstream effector, the ribosomal protein S6 kinase 1 (S6K1) [92]. Furthermore, exercise upregulates the expression of receptor proteins specifically mediating mitochondrial autophagy, such as BNIP3L and Parkin [93,94]. Collectively, these alterations facilitate the recognition and engulfment of damaged mitochondria by autophagosomes, culminating in their degradation through fusion with lysosomes. This restores normal mitochondrial autophagy flux, thereby preventing the vicious cycle of toxic protein accumulation and oxidative stress.

**Table 3:** Key Experimental Evidence for the Dynamic Equilibrium of Mitochondrial Remodelling and Autophagic Flow.

References	Experimental Subjects/Models	Exercise Intervention Programme	Key Research Findings	Core Pathways/Molecules Involved
Xie et al. [89]	D-galactose-induced aging in C57BL/6 mice	8-week treadmill training Programme	Downregulation of hippocampal Drp1 protein expression, alongside upregulation of Mfn2 and TFAM expression, improves spatial learning and memory abilities in the water maze test.	Drp1, Mfn2 (dynamic)
Jin et al. [86]	D-gal-induced aging in SD rats	6-week swimming training programme	Significant increases in hippocampal cAMP levels and PKA expression; Improved synaptic plasticity and spatial learning and memory deficits.	cAMP/PKA (kinetic upstream)
Kim et al. [87]	Cell senescence model	Mechanism Research	PKA-mediated phosphorylation of Drp1 (Ser637) inhibits its mitochondrial translocation and fission activity, thereby delaying cellular senescence.	PKA/Drp1
Qin et al. [93]	Female aged C57 mice	9-week self-directed treadmill exercise programme	Expression of Bnip3L and Atg7, molecules associated with mitochondrial autophagy in the cerebral cortex, is upregulated; This improves neuromuscular function and exercise endurance.	Bnip3L, Atg7 (autophagy)
Luo et al. [95]	Aged rats	10-week swimming training programme	Elevated LC3-II/LC3-I ratio and Parkin levels, alongside reduced p62 levels, mitigate age-related cognitive decline.	LC3, Parkin, p62 (autophagy pathway)
Pan et al. [94]	Aged C57 mice	12-week swimming training programme	Increased cortical LC3-II/LC3-I ratio, Parkin and BNIP3L expression; enhanced AMPK phosphorylation, reduced mTOR and S6K phosphorylation; improved learning and memory.	AMPK/mTOR/S6K, Parkin, BNIP3L (autophagy regulation)
Liu et al. [96]	Aged SD rats	10-week moderate-intensity treadmill training programme	Striatal pAMPKα1, Beclin-1 and LC3-II expression was significantly elevated.	AMPK, Beclin-1 (autophagy initiation)

Thus, exercise remodels the mitochondrial quality control system not through a single pathway, but via a synergistic network. On one hand, it precisely regulates Drp1 activity via the cAMP/PKA signaling axis, inhibiting excessive fission while promoting fusion protein expression to restore a healthy mitochondrial network architecture. On the other hand, it releases autophagy inhibition through the AMPK/mTOR signaling axis and upregulates specific autophagy receptors, thereby accelerating the recognition and clearance of damaged mitochondria. These two processes complement each other: healthy dynamics provide autophagy with correct targets for clearance (i.e., damaged segments isolated by moderate fission), while efficient autophagy clears damaged mitochondrial components for the dynamic process, allowing the establishment of a newly fused, healthy mitochondrial network. This systemic optimisation of the mitochondrial “morphology–function–renewal” cycle constitutes the indispensable cellular and molecular basis for exercise’s capacity to enhance energy metabolism and synaptic function in the aging brain, ultimately delaying cognitive decline.

### 5.3. Exercise-Induced Systemic Adaptations

The neuroprotective effects of exercise extend far beyond direct stimulation of the brain. A growing body of evidence indicates that regular physical activity induces robust systemic adaptations, indirectly and persistently supporting brain health and cognitive function through a dynamic bidirectional communication system termed the “muscle–brain axis” [97,98]. In this process, skeletal muscle during exercise functions as a crucial endocrine organ, releasing a series of factors into the circulatory system. These factors either directly cross the blood–brain barrier or transmit signals via receptors on the barrier surface, thereby promoting neurogenesis, enhancing synaptic plasticity, and optimising mitochondrial function in the distant brain (Table 4).

#### 5.3.1. Muscle Factor Signaling: Neurotrophic Support and Metabolic Regulation

Exercise significantly upregulates the synthesis and release of multiple bioactive molecules from skeletal muscle. Among these, insulin-like growth factor-1 (IGF-1) plays a pivotal role. Research confirms that an 8-week treadmill exercise programme significantly increases IGF-1 expression in the hippocampal region of 17-month-old male mice [99]; similarly, a 4-week treadmill regimen elevates IGF-1 levels in the substantia nigra of 18-month-old rats [100]. Animal studies suggest that circulating IGF-1 may cross the blood–brain barrier, potentially via transporter-mediated mechanisms, and activate the IGF-1R/PI3K/Akt survival signaling pathway within neurons. This pathway not only promotes neuronal survival and inhibits apoptosis but also synergises with other brain pathways such as AMPK/SIRT1/PGC-1α to collectively enhance mitochondrial biogenesis and energy metabolism, thereby delaying brain aging [98,101]. Beyond IGF-1, animal studies have shown that exercise may increase the expression and release of several myokines including irisin and cathepsin B. These factors have been suggested to enter the circulation and promote hippocampal BDNF expression and neurogenesis, potentially contributing to the neuroprotective effects of exercise [83].

#### 5.3.2. Systemic Signaling via Extracellular Vesicles

Extracellular vesicles are emerging candidates for exercise-induced muscle-to-brain communication; however, their cargo specificity, brain targeting, and causal role in mitochondrial adaptation remain to be clarified. For instance, exercise-derived EVs may contain miRNAs regulating neuroplasticity, mitochondrial dynamics, or inflammatory responses. Upon uptake by cerebral vascular endothelial cells or neurons, these vesicles release their instructions, thereby modulating gene expression in the brain at the post-transcriptional level and indirectly supporting neural health and mitochondrial function [102].

#### 5.3.3. Lactate: From Metabolic Waste to Multifunctional Signaling Molecule

Lactate produced during exercise should be considered a metabolic intermediate and signaling molecule rather than merely a waste product. During exercise, elevated blood lactate levels enable its passage across the blood–brain barrier via monocarboxylate transporters. Within the brain, lactate may be oxidised and utilised by astrocytes and neurons as an alternative energy source, supporting neuronal metabolic demands—particularly during periods of heightened energy requirements [97]. More importantly, experimental studies suggest that lactate may also function as a signaling molecule by activating neuronal receptors such as GPR81, thereby initiating downstream pathways associated with neuroplasticity, angiogenesis, and antioxidant responses, which may contribute to neuroprotective effects [59]. In aged mouse models, long-term high-intensity interval training (HIIT) has been shown to significantly elevate brain lactate levels in association with improved cognitive performance, further supporting the potential signaling role of lactate in exercise-induced neuroprotection [97].

In summary, exercise-induced protection of brain health constitutes an integrated adaptive process involving multiple organs. Skeletal muscle establishes a robust “muscle–brain axis” communication pathway by releasing neurotrophic factors (such as IGF-1), signaling vesicles (EVs), and metabolites (such as lactate). These circulating factors act upon the brain in complementary ways: IGF-1 and others deliver classical growth and survival signals; extracellular vesicles (EVs) may facilitate intercellular transfer of regulatory molecules and signaling information; and lactate simultaneously supplies immediate energy substrates and activates protective signaling pathways. This exercise-induced systemic adaptation complements exercise’s direct effects on the brain, collectively contributing to the biological basis underlying exercise-mediated neuroprotection.

However, despite increasing interest in the muscle–brain axis, several important questions remain unresolved. Current evidence regarding exercise-induced circulating factors is still largely derived from animal studies, while direct mechanistic validation in humans remains limited. In particular, the extent to which myokines, extracellular vesicles, and metabolites such as lactate can cross the blood–brain barrier and exert sustained effects on neuronal mitochondrial function is not yet fully understood. Moreover, findings across studies are sometimes inconsistent due to differences in exercise modalities, intervention duration, age, and baseline metabolic status. Future studies should therefore focus on clarifying the causal relationships, tissue specificity, and translational relevance of these systemic signaling pathways in human brain aging [97,98,102].

## 6. Optimising Exercise Interventions to Delay Brain Aging

Exercise, as a non-pharmacological intervention to delay brain aging, has been extensively supported by evidence for its benefits. However, the efficacy of exercise interventions is modulated by multiple factors including exercise type, intensity, frequency, and individual characteristics [7]. Different exercise modalities exert distinct effects on brain structure, mitochondrial function, and neuroplasticity through diverse physiological and molecular mechanisms [7]. Therefore, understanding the specific characteristics and potential limitations of different exercise strategies is essential for developing effective interventions against brain aging.

### 6.1. Moderate-Intensity Continuous Training: The Cornerstone for Enhancing Basal Metabolism and Neuroplasticity

Moderate-intensity continuous training (e.g., brisk walking, jogging, swimming) represents the most thoroughly researched and safest foundational programme. It has been shown to improve cardiovascular health, increase cerebral blood flow, and enhance mitochondrial function while promoting neuroplasticity through multiple pathways including AMPK/PGC-1α and BDNF [103,104]. Its core mechanism lies in the sustained, stable enhancement of cardiovascular function and cerebral blood perfusion, thereby supplying the brain with ample oxygen and nutritional substrates [7]. At the molecular level, it effectively activates the AMPK/PGC-1α signaling axis, promotes mitochondrial biogenesis, and upregulates brain-derived neurotrophic factor (BDNF) levels, consequently enhancing synaptic plasticity and neuronal survival [7]. A recent study further suggested that moderate-intensity aerobic training may reduce neuronal apoptosis and improve learning and memory, potentially through miR-21-3p-related mechanisms [97]. A preliminary 2024 study comparing exercise patterns among community-dwelling older adults found that six months of moderate-intensity aerobic exercise significantly improved executive function, an effect correlated with increased surface area in the right dorsolateral prefrontal cortex [105].

### 6.2. High-Intensity Interval Training: A Time-Efficient Metabolic and Signaling Stimulus

High-Intensity Interval Training (HIIT) is characterised by its time efficiency and profound disruption of the metabolic system. HIIT rapidly induces substantial lactate production in skeletal muscle. Lactate serves not only as an energy substrate but also as a crucial signaling molecule capable of traversing the blood–brain barrier to directly influence neurons. It may promote neuroplasticity and angiogenesis by activating pathways such as PGC-1α, potentially offering unique advantages in improving executive function and cerebral metabolism in older individuals [97,106]. A recent animal study proposed a potentially novel cross-organ mechanism underlying the neuroprotective effects of HIIT, suggesting that mitochondria originating from exercised skeletal muscle may be transported via circulating platelets to the brain, where they could contribute to white matter repair and functional recovery following cerebral ischemia [107]. However, this hypothesis is currently based on a single mouse study, and it remains unclear whether similar mechanisms occur under physiological aging conditions or in humans. Therefore, the concept of exercise-induced “mitochondrial migration” should be regarded as preliminary and requires further experimental and translational validation.

### 6.3. Resistance Training: A Pillar in Combating Sarcopenia and Preserving Survival Signaling

The contribution of resistance training (strength training) to brain health is frequently underestimated. Its most direct effect lies in counteracting age-related muscle loss (sarcopenia). Muscle tissue functions not only as a motor organ but also as a significant endocrine organ. Resistance training significantly increases muscle mass and strength while elevating circulating insulin-like growth factor-1 (IGF-1) levels. IGF-1 crosses the blood–brain barrier, activating the IGF-1R/PI3K/Akt survival signaling pathway within neurons. This pathway is crucial for sustaining neuronal survival and inhibiting apoptosis [98,108,109,110]. Moreover, greater muscle mass correlates with elevated basal metabolic rates and improved systemic glucose-lipid metabolism regulation, indirectly fostering a healthier systemic environment for the brain [110]. Thus, resistance training constitutes a pivotal peripheral support mechanism for central nervous system health. Nevertheless, evidence regarding the direct effects of resistance training on brain mitochondrial quality control remains relatively limited compared with aerobic exercise.

### 6.4. Mind–Body Practices and Combined Training: Multidimensional Synergistic Effects

Mind–body practices such as Tai Chi and yoga, alongside combined training protocols integrating aerobic, resistance, and balance exercises, represent more holistic intervention approaches. These approaches not only confer combined physiological benefits but also improve psychological well-being through enhanced mind–body awareness, breathing regulation, and mindfulness [111,112]. This effectively reduces chronic stress hormone levels (such as cortisol), as prolonged exposure to elevated cortisol exerts clear neurotoxic effects on hippocampal neurons and mitochondria [113,114,115]. By alleviating stress, improving sleep, and elevating mood, mind–body exercises cultivate an optimised internal microenvironment conducive to neuroplasticity and neuronal health. Their mechanisms may involve coordinated physiological and psychological adaptations. However, empirical research in this area remains relatively scarce and warrants further strengthening [116]. Moreover, the molecular mechanisms underlying these benefits remain insufficiently characterised.

Nevertheless, findings regarding the optimal exercise modality and intensity for delaying brain aging remain inconsistent. While moderate-intensity aerobic exercise is generally considered safe and effective, some studies suggest that high-intensity exercise may induce excessive oxidative stress or inflammatory responses in vulnerable older individuals [117]. In contrast, other studies report superior metabolic and cognitive benefits following high-intensity interval training [97,106]. These discrepancies may be related to differences in age, sex, genetic background, baseline fitness, and disease status. Furthermore, variations in exercise protocols and outcome measures across studies complicate direct comparisons. In addition, some individuals may exhibit limited responsiveness to exercise interventions (exercise non-responders) [118], although the mechanisms underlying this phenomenon in brain aging remain poorly understood. Therefore, a “one-size-fits-all” exercise prescription for brain aging is unlikely to be appropriate. Taken together, these findings highlight the importance of developing personalised and adaptable exercise strategies based on individual physiological characteristics and aging trajectories.

## 7. Discussion and Future Directions

Based on the above considerations, exercise interventions for delaying brain aging should extend beyond single-modality approaches and move towards more individualised and integrative strategies. In practice, factors such as age, baseline health status, cognitive function, and prior exercise experience need to be taken into account when designing interventions. Initiating programmes with moderate-intensity continuous training (MICT) or mind–body exercise may help improve adherence, with gradual progression in intensity or complexity over time [119]. The integration of genomic or metabolic information with clinical characteristics may further support more precise exercise prescription, although such approaches are still in their early stages.

Multimodal exercise strategies may offer broader benefits than single-mode interventions, given the partially complementary effects of different exercise types. Combined approaches that include aerobic, resistance, and neuromuscular training have been associated with improvements in both physical and cognitive outcomes [120]. There is also increasing interest in whether exercise can be combined with other non-pharmacological interventions, such as nutritional supplementation, cognitive training, or non-invasive brain stimulation. These interventions may act through partially overlapping pathways, including SIRT1/PGC-1α signaling.

The optimisation of exercise dose is another important but not yet fully resolved issue. While general recommendations (e.g., 150 min of moderate-intensity or 75 min of vigorous-intensity activity per week) provide a useful reference, the relationship between exercise parameters and cognitive outcomes is unlikely to be linear [7]. An inverted J-shaped pattern has been suggested, indicating that both insufficient and excessive exercise may be less effective [121]. In addition, very-high-intensity exercise may increase oxidative stress or injury risk in certain populations. Further work is therefore needed to clarify dose–response relationships across different age groups and exercise modalities.

Several limitations should also be considered. A large proportion of the mechanistic evidence discussed here is derived from animal studies, while direct evidence in humans remains relatively scarce. In particular, the role of mitochondrial quality control in exercise-related brain aging has not been fully established in clinical populations. This limits the extent to which current findings can be translated, especially given potential differences between species in brain structure, metabolism, and responsiveness to exercise [7,119]. In addition, variability between individuals may further influence outcomes. Greater emphasis should be placed on well-designed human trials, including longitudinal and interventional approaches, as well as from the identification of peripheral biomarkers that can reflect mitochondrial adaptations in a non-invasive manner.

Importantly, increasing evidence suggests that individual responses to exercise interventions may vary substantially depending on genetic background, metabolic status, and baseline mitochondrial function [118]. Individuals carrying neurodegenerative disease-related risk factors may exhibit distinct patterns of mitochondrial dysfunction, neuroinflammation, and reduced neuroplasticity during brain aging, potentially influencing their responsiveness to exercise interventions [122]. Moreover, the phenomenon of exercise non-responders has been increasingly recognised in metabolic and cardiovascular research, although its relevance and underlying mechanisms in brain aging remain poorly understood [118]. Addressing these issues will be important for identifying biomarkers capable of predicting individual responsiveness and developing personalised exercise prescriptions targeting specific aging trajectories and risk profiles.

Overall, current evidence suggests that exercise may influence multiple aspects of mitochondrial quality control and contribute to the maintenance of brain health during aging. However, substantial gaps remain regarding the optimal exercise parameters, individual variability, and the translational relevance of mechanistic findings from animal models to humans.

## 8. Conclusions

Brain aging is closely associated with progressive disruption of mitochondrial quality control, including impairments in mitochondrial biogenesis, dynamics, mitophagy, and energy metabolism. These alterations contribute to mitochondrial dysfunction, energy deficits, and cognitive decline. Exercise, as a multi-target non-pharmacological intervention, appears to modulate these processes through pathways such as AMPK/SIRT1/PGC-1α, thereby supporting mitochondrial homeostasis and neuroprotection. In addition, exercise-induced systemic adaptations involving myokines, extracellular vesicles, and metabolites such as lactate may further contribute to brain health through muscle–brain communication.

Although increasing evidence supports the beneficial effects of exercise on brain aging, important mechanistic and translational questions remain unresolved, particularly regarding individual variability and the applicability of findings from animal models to humans. Nevertheless, exercise-based interventions remain a promising strategy for promoting healthy brain aging and reducing the burden of age-related neurodegenerative disorders.

## Figures and Tables

**Table 1:** Multidimensional Evidence of Mitochondrial Energy Metabolism Dysfunction in Brain Aging.

Functional Impairment Level	Core Findings	Potential Intervention Targets/Strategies	Representative Studies
Decline in mitochondrial energy metabolism	Animal studies consistently report reduced ATP levels and mitochondrial membrane potential, whereas studies of human aging brain tissue demonstrate alterations in NAD+/NADH homeostasis and mtDNA integrity.	Enhance the overall cellular energy state, such as by supplementing NAD+ precursors and activating the AMPK pathway.	[52,53,72,73,74,75,76,77,78]
TCA cycle enzyme dysfunction	Decreased activity/expression of key enzymes such as α-ketoglutarate dehydrogenase, cis-aconitase, and malate dehydrogenase leads to circulatory stasis and loss of metabolic flux.	Targeted enhancement of TCA cycle enzyme activity (e.g., via gene therapy); Utilisation of metabolic bypass substrates (e.g., dimethyl succinate) to circumvent enzyme activity barriers.	[68,69]
Impaired electron transport chain and oxidative stress	Impaired function of the electron transport chain complex is accompanied by excessive ROS production, reduced membrane potential, and diminished oxidative phosphorylation efficiency.	Apply mitochondrial-targeted antioxidants; Directly and controllably enhance mitochondrial membrane potential and function via chemogenetic tools.	[70,71]
The decline in the “on-demand energy supply” mechanism	Aging leads to a weakening of the coupling mechanism whereby neural activity drives mitochondrial gene transcription, preventing mitochondria from responding to synaptic energy demands through adaptive biogenesis.	Enhance cognitive activity (mental exercise); restore this coupling mechanism through molecular tools (such as expressing constitutively activated mitochondrial CREB).	[22]
Metabolic Mode Shift	Energy metabolism shifts from efficient oxidative phosphorylation to inefficient glycolysis, with a loss of metabolic flexibility.	Through interventions such as aerobic exercise, reverse metabolic patterns and enhance mitochondrial oxidative capacity.	[67,68]

**Table 2:** Key Experimental Evidence for Exercise-Promoted Mitochondrial Biogenesis in the Brain.

References	Research Subject/Model	Exercise Intervention Programme	Key Findings (Related to Mitochondrial Biogenesis)	Core Signaling Molecules Involved
Belviranli et al. [80]	20-month-old female Wistar rats	90-day self-guided treadmill exercise programme	The expression level of PGC-1α protein in hippocampal tissue was significantly elevated.	PGC-1α
E et al. [81]	18-month-old male C57BL/6 mice	8-week treadmill Training programme (super-lactate threshold intensity)	Increased expression of PGC-1α, NRF1 and NRF2 proteins in the hippocampus, alongside elevated levels of BDNF.	PGC-1α, NRF1/2, BDNF
Liu et al. [84]	18-month-old C57BL/6N mice	5-week ladder resistance training	Increased expression of hippocampal PGC-1α and BDNF, accompanied by improvements in mitochondrial morphology and function (with a reduction in the proportion of abnormal mitochondria).	PGC-1α, BDNF
De Sousa et al. [85]	18-month-old male C57BL/6J mice	2-week intensive swimming programme	Proteins associated with mitochondrial biogenesis and survival were enhanced in the hippocampus, with the mechanism linked to activation of the SIRT1/PGC-1α signaling pathway.	SIRT1/PGC-1α
Wrann et al. [83]	Animal models and cell experiments	Exercise training paradigm (as previously described)	This study provides evidence for the skeletal muscle exercise-induced PGC-1α/FNDC5/irisin pathway, which may mediate the distal effects of exercise on hippocampal BDNF upregulation, suggesting the existence of an exercise-induced muscle–brain communication axis.	PGC-1α/FNDC5/BDNF

**Table 4:** Key Research Evidence on Exercise’s Impact on Brain Health Through Systemic Adaptations.

References	Systemic Factors/Mechanisms Involved	Experimental Subjects/Models	Exercise Intervention Programme	Key Research Findings (Related to Brain Health)
Lee et al. [99]	Insulin-like growth factor-1 (IGF-1)	17-month-old male C57BL/6J mice	8-week treadmill training programme	Increased IGF-1 expression in the hippocampus, alongside enhanced cognitive function, accompanied by amplified antioxidant and neurotrophic responses.
Munoz et al. [100]	Insulin-like growth factor-1 (IGF-1)	18-month-old SD rats	4-week treadmill training programme	Increasing IGF-1 levels in the substantia nigra while simultaneously ameliorating other molecular alterations associated with aging in this brain region.
Lin et al. [101]	IGF-1/PI3K/Akt pathway	D-gal-induced aging in male rats	8-week swimming training programme	Increased levels of hippocampal p-IGF1R, p-PI3K and p-Akt proteins, concurrent with improved mitochondrial function and anti-apoptotic effects.
Wrann et al. [83]	PGC-1α–FNDC5/irisin–BDNF signaling axis	Animal models and cell experiments	Exercise training paradigm (as previously described)	Exercise generates irisin via the muscle PGC-1α/FNDC5 pathway, which may mediate the upregulation of hippocampal BDNF.
Lei et al. [97]	Lactate	20–22-month-old aged mice	7-week high-intensity interval training (HIIT)	HIIT significantly improved cognitive performance in aged mice, with the study highlighting the potential role of exercise-induced lactate in promoting brain neuroplasticity.

## Data Availability

No new data were created or analyzed in this study. Data sharing is not applicable to this article.
